# The complete mitochondrial genome of *Aeolothrips indicus* Bhatti, 1964 (Thysanoptera: Thripidae)

**DOI:** 10.1080/23802359.2021.1970647

**Published:** 2021-08-31

**Authors:** Avas Pakrashi, Kaomud Tyagi, Vikas Kumar

**Affiliations:** a Centre for DNA Taxonomy, Molecular Systematics Division, Zoological Survey of India, Kolkata, India; b Department of Zoology, University of Calcutta, Kolkata, India

**Keywords:** Mitogenome, Aeolothripidae, *Aeolothrips indicus*

## Abstract

Here, we have generated the complete mitochondrial sequence of *Aeolothrips indicus* Bhatti, 1964. So far, this is the first largest mitogenome with 17,042 bp length in order Thysanoptera. It includes 13 protein-coding genes, 22 transfer RNA genes, and two ribosomal RNA genes along with three non-coding regions. AT composition of *A. indicus* is 72.5% (37.7% A and 34.8% T) and GC 27.5% (15.6% C and 11.9% G). The constructed phylogeny revealed the monophyly of family Aeolothripidae in the order Thysanoptera. The data would provide further insight into the evolution and phylogeny of the order Thysanoptera.

The family Aeolothripidae of order Thysanoptera comprises of less than 4% of thrips diversity worldwide, including 218 extant species in 24 genera (ThripsWiki 2021). Most of species in this family are phytophagous in nature, while a few are monophagous like *Indothrips bhushani* Bhatti is restricted to neem flowers (*Azadirachta indica*) for a very short period of time in India (Tyagi et al. 2008). They are also known to be obligate or facultative predator on minute arthropods (Mound and Marullo 1998). The genus *Aeolothrips* was first described by Haliday in 1836 with type *Aeolothrips albicincta* Haliday. This genus is known by 113 species across the world (ThripsWiki 2021), of which seven species reported from India (Tyagi and Kumar 2016). *Aeolothrips indicus* was described by Bhatti in 1964 (Bhatti 1964), and also known to be endemic to India. This is the second mitogenome for the family Aeolothripidae and first largest genome in order Thysanoptera.

Specimen of *A. indicus* was collected from the flowers and leaves of *Mangifera indica* in March 2020 from Nainital (29.28N, 79.27E), Uttarakhand, India. The nondestructive DNA extraction from a single specimen was done with the DNeasy Blood and Tissue Kit (QIAGEN, Hilden, Germany). Voucher specimen with registration no. 11139/H17 was deposited in the National Zoological Collections (NZC) at the Centre for DNA Taxonomy, Molecular Systematics Division, Zoological Survey of India, Kolkata, India (collection in-charge, Dr. Vikas Kumar, kumar.vikas@zsi.gov.in). The sequencing was done in Illumina platform (NovaSeq 6000) with 2 × 150 base pair chemistry and the raw reads were assembled by GetOrganelle software (version 1.7.4) (Jin et al. 2020) using COI sequence of this species as a seed. The annotation of the gene boundaries was done by MITOS Web Server (Bernt et al. 2013), ORF Finder (https://www.ncbi.nlm.nih.gov/orffinder), BLASTn, and BLASTp. Further, these boundaries were confirmed with the alignment of previously available thrips mitogenomes.

*A. indicus* (17,042 bp) mitogenome includes 37 genes with 13 protein-coding genes (PCGs), 22 transfer RNAs (tRNAs), two ribosomal RNAs (rRNAs), and three putative control regions. This is the second mitogenome in the order Thysanoptera which was reported with three control regions (Tyagi et al. 2020). Most of the genes were located on the majority strand except *nad5*, *nad4*, *nad4L*, *trnH*, and *trnP*. The AT content of the genome was 72.5% (37.7% of A and 34.8% of T) and GC 27.5% (11.9% of G and 15.6% of C). *atp8*, *cox2, nad2, nad4L* were used as ATA start codon; *cox1, cox3, nad1, nad4, nad5, nad6* by ATT; and *atp6, cytb, nad3* by ATG. TAA stop codon was used by all the PCGs with few exceptions, like TAG stop codon was used by *atp8, nad2, nad4L, nad5*. tRNAs were ranging from 74 bp (*trnR*) to 61 bp (*trnS1*) with typical cloverleaf secondary structure. The length of *rrnL* and *rrnS* was 1325 bp and 804 bp, respectively. Five overlapping regions (1–10 bp with a total of 18 bp) and 23 intergenic spacer regions (1–78 bp with a total of 330 bp) were detected in the mitogenome.

The TranslatorX tool (Abascal et al. 2010) was used for the PCGs alignment and SequenceMatrix v1.8 (Vaidya et al. 2011) for PCGs concatenation. The GTR + I+G was detected as a best fit model in PartitionFinder version 2.1.1 with BIC criterion (Lanfear et al. 2017). Mr.Bayes ver. 3.2 (Ronquist et al. 2012) was used for the construction of the Bayesian inference (BI) and IQ tree web server (http://iqtree.cibiv.univie.ac.at/) for maximum-likelihood (ML) phylogenetic trees. *Alloeorhynchus bakeri* (Hemiptera) sequence (GenBank accession HM235722) was used as an out group for the phylogeny. The similar topology was revealed by both phylogenetic methods and their support values were superimposed on a BI tree in Figure 1. Both the phylogeny reflected the monophyly of the family Aeolothripidae with high support.

**Figure 1.**
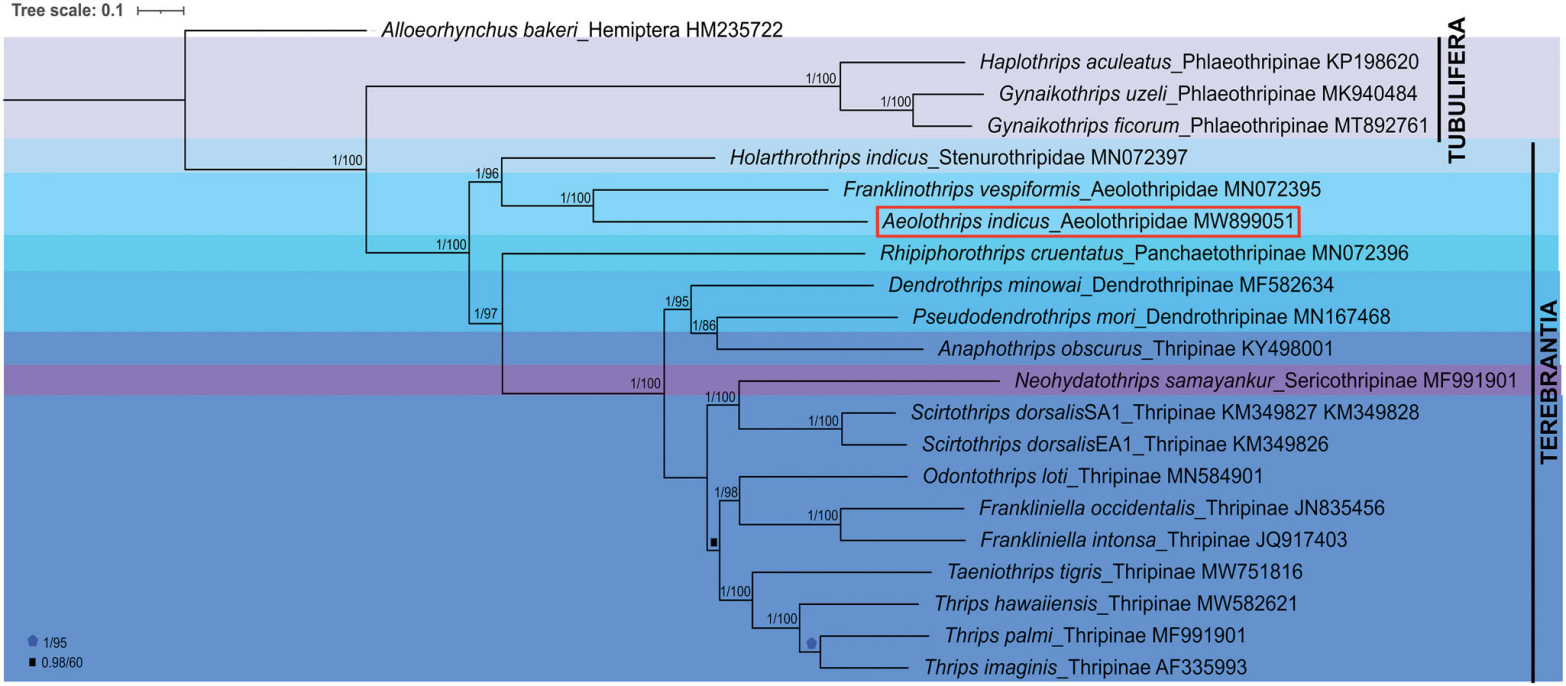
Bayesian inference using 13 PCGs and the posterior probabilities (pp) and bootstrap support (bs) are superimposed. The GenBank accession numbers of all the studied species are also provided. The hemipteran *Alloeorhynchus bakeri* was used as an outgroup.

## Data Availability

The genome sequence data that support the findings of this study are openly available in GenBank of NCBI at under the accession no. MW899051. The associated BioProject, SRA, and Bio-Sample numbers are PRJNA752518, SRR15358082, and SAMN20600165, respectively.

## References

[CIT0001] Abascal F, Zardoya R, Telford MJ. 2010. TranslatorX: multiple alignment of nucleotide sequences guided by amino acid translations. Nucleic Acids Res. 386:13. 10.1093/nar/gkq291 PMC2896173 20435676

[CIT0002] Bernt M, Donath A, Jühling F, Externbrink F, Florentz C, Fritzsch G, Pütz J, Middendorf M, Stadler PF. 2013. MITOS: improved de novo metazoan mitochondrial genome annotation. Mol Phylogenet Evol. 69(2):313–319. 22982435 10.1016/j.ympev.2012.08.023

[CIT0003] Bhatti JS. 1964. Studies on the Indian species of the genus Aelothrips Hal. Bull Entomol. 5:17–23.

[CIT0004] Jin JJ, Yu WB, Yang JB, Song Y, Depamphilis CW, Yi TS, Li DZ. 2020. GetOrganelle: a fast and versatile toolkit for accurate de novo assembly of organelle genomes. Genome Biol. 21(1):241. 32912315 10.1186/s13059-020-02154-5 PMC7488116

[CIT0005] Lanfear R, Frandsen PB, Wright AM, Senfeld T, Calcott B. 2017. Partitionfinder 2: new methods for selecting partitioned models of evolution for molecular and morphological phylogenetic analyses. Mol Biol Evol. 34(3):772–773. 28013191 10.1093/molbev/msw260

[CIT0006] Mound LA, Marullo R. 1998. Biology and identification of Aeolothripidae (Thysanoptera) in Australia. Invert Syst. 12(6):929–950.

[CIT0007] Ronquist F, Teslenko M, Mark PV, Ayres DL, Darling A, Höhna S, Larget B, Liu L, Suchard MA, Huelsenbeck JP. 2012. MrBayes 3.2: efficient Bayesian phylogenetic inference and model choice across a large model space. Syst Biol. 61(3):539–542. 22357727 10.1093/sysbio/sys029 PMC3329765

[CIT0008] ThripsWiki. 2021. ThripsWiki—providing information on the World's thrips; [accessed 2021 Apr 15]. https://thrips.info/wiki/.

[CIT0009] Tyagi K, Chakraborty R, Cameron SL, Sweet AD, Chandra K, Kumar V. 2020. Rearrangement and evolution of mitochondrial genomes in Thysanoptera (Insecta). Sci Rep. 10(1):695. 31959910 10.1038/s41598-020-57705-4 PMC6971079

[CIT0010] Tyagi K, Kumar V. 2016. Thrips (Insecta: Thysanoptera) of India—an updated checklist. Halteres. 7:64–98.

[CIT0011] Tyagi K, Mound L, Kumar V. 2008. Sexual dimorphism among Thysanoptera Terebrantia, with a new species from Malaysia and remarkable species from India in Aeolothripidae and Thripidae. Insect Syst Evol. 39(2):155–170.

[CIT0012] Vaidya G, Lohman DJ, Meier R. 2011. SequenceMatrix: concatenation software for the fast assembly of multi-gene datasets with character set and codon information. Cladistics. 27(2):171–180. 10.1111/j.1096-0031.2010.00329.x 34875773

